# Association Between Parenting and School Refusal Among Elementary School Children in Japan: Results From A-CHILD Longitudinal Study

**DOI:** 10.3389/fped.2021.640780

**Published:** 2021-03-26

**Authors:** Yoshifumi Fukuya, Takeo Fujiwara, Aya Isumi, Satomi Doi, Manami Ochi

**Affiliations:** ^1^Department of Global Health Promotion, Tokyo Medical and Dental University, Tokyo, Japan; ^2^Department of Health and Welfare Services, National Institute of Public Health, Saitama, Japan

**Keywords:** school refusal, parenting, parent-child interaction, child maltreatment, mental health, prevention

## Abstract

**Objective:** The aim of this study was to examine the association between parenting, including the parent–child interaction and child maltreatment in the first grade (6–7 years old), and school refusal in the second (7–8 years old) and fourth (9–10 years old) grades among elementary school children in Japan.

**Methods:** Data were from the Adachi Child Health Impact of Living Difficulty (A-CHILD) longitudinal study conducted in 2015, 2016, and 2018 in Adachi City, Tokyo, Japan. A questionnaire was distributed to all the first-grade school children (*N* = 5,355) in 2015. Of the total 4,291 valid children (response rate: 80.1%), 3,590 and 3,070 children were followed up to the second and fourth grades, respectively. Caregivers responded to the questionnaire on the parent–child interaction and child maltreatment, including neglect, physical abuse, and psychological abuse in the first grade and school refusal in the second and fourth grades. We conducted multiple imputation for missing data. Multivariate logistic regression model was used for this analysis adjusting for child mental health in the first grade and sociodemographic characteristics.

**Results:** Prevalence of school refusal was 1.8% (*n* = 64) in the second grade and 2% (*n* = 60) in the fourth grade. We found no association of the parent–child interaction and child maltreatment in the first grade and with school refusal in the second and fourth grades, respectively, after adjusting for covariates.

**Conclusions:** Parenting, such as the parent–child interaction and child maltreatment, may not be associated with school refusal among elementary school children. Further longitudinal research is needed to elucidate other factors, such as peer relationships and school environment, which can affect school refusal.

## Introduction

School refusal is one of the important school-related problems. School refusal is defined as child-motivated refusal to attend school or difficulties remaining in the classroom for an entire day with the presence of emotional upset, such as anxiety and depression (1, 2). An estimated 0.5–5% of children show school refusal in the United States (3, 4), Venezuela (5), and India (6). School refusal has negative impacts on the emotional and social development and academic performance in childhood (7–9) and can lead to psychiatric illnesses and occupational and marital problems in adulthood (10, 11). Hence, it is crucial to elucidate the risk factors for school refusal to prevent these adverse consequences.

Parenting may be associated with school refusal. As one form of parenting, the parent–child interaction plays an essential role in the socioemotional and educational development of children. In daily life, parents interact with their children in various ways: from communicating and doing activities together (e.g., playing and cooking) to getting involved in their education (e.g., helping their schoolwork and talking about school), which affects mental health and school performance of children. For example, previous studies reported that parent–child communication had beneficial effects on well-being of children (12) and fewer behavior problems (13). Moreover, cooking with children is associated with the responsibility and self-esteem (14). Research has also shown that the parental involvement in cooking activities evokes positive emotions and feeling in control (15) among children. Furthermore, the parental involvement in children's education outside school affects academic achievements and attitudes and the motivation toward school among children (16–19). Prior studies have shown a positive association of the parental involvement at home with their school performance and attendance among children (16, 20, 21). Research has also reported the association of less parental involvement at home with school attendance problems among children, such as truancy and dropped out (22, 23). Given the psychosocial and academic benefits of the parent–child interaction, it may function as one of the protective factors for school refusal among children. However, no published study so far examined the association between the parent–child interaction and school refusal.

As a deviant form of parenting, child maltreatment has adverse impacts on emotional and social development of children. Child maltreatment also causes psychosomatic symptoms, mental illness, low self-esteem, antisocial problems, and impaired self-regulation in children (24). Further, prior research has indicated an association between child maltreatment and school absenteeism among adolescents (25, 26). Therefore, it is reasonable to consider that child maltreatment could be a risk factor for school refusal. However, no studies have focused on the relationship between child maltreatment and school refusal. For preventive interventions for school refusal, there is a need to assess whether maltreated children have an increased risk of school refusal.

To date, few empirical studies of school refusal have been conducted in prospective designs. A longitudinal study is needed to investigate the relationships between potential risk factors and school refusal for causality. Moreover, according to the Ministry of Education, Culture, Sports, Science, and Technology of Japan, the number of chronic school absenteeism, which can be linked to a negative outcome of school refusal, in the second, fourth, and sixth grades has increased by 1.5, 3.5, and 6.1 times, respectively, compared with the first grade (27). Accordingly, there is an urgent need to address this attendance problem and to identify risk and protective factors for school refusal before the problem gets worse for the prevention. The aim of this study was to examine the association between parenting and school refusal in a general population of elementary school children in Japan using a longitudinal dataset. We then focused on the impacts of the parent–child interaction and child maltreatment as the indicators of parenting in the first grade on school refusal in the second and fourth grades.

## Materials and Methods

### Participants

**Figure 1:**
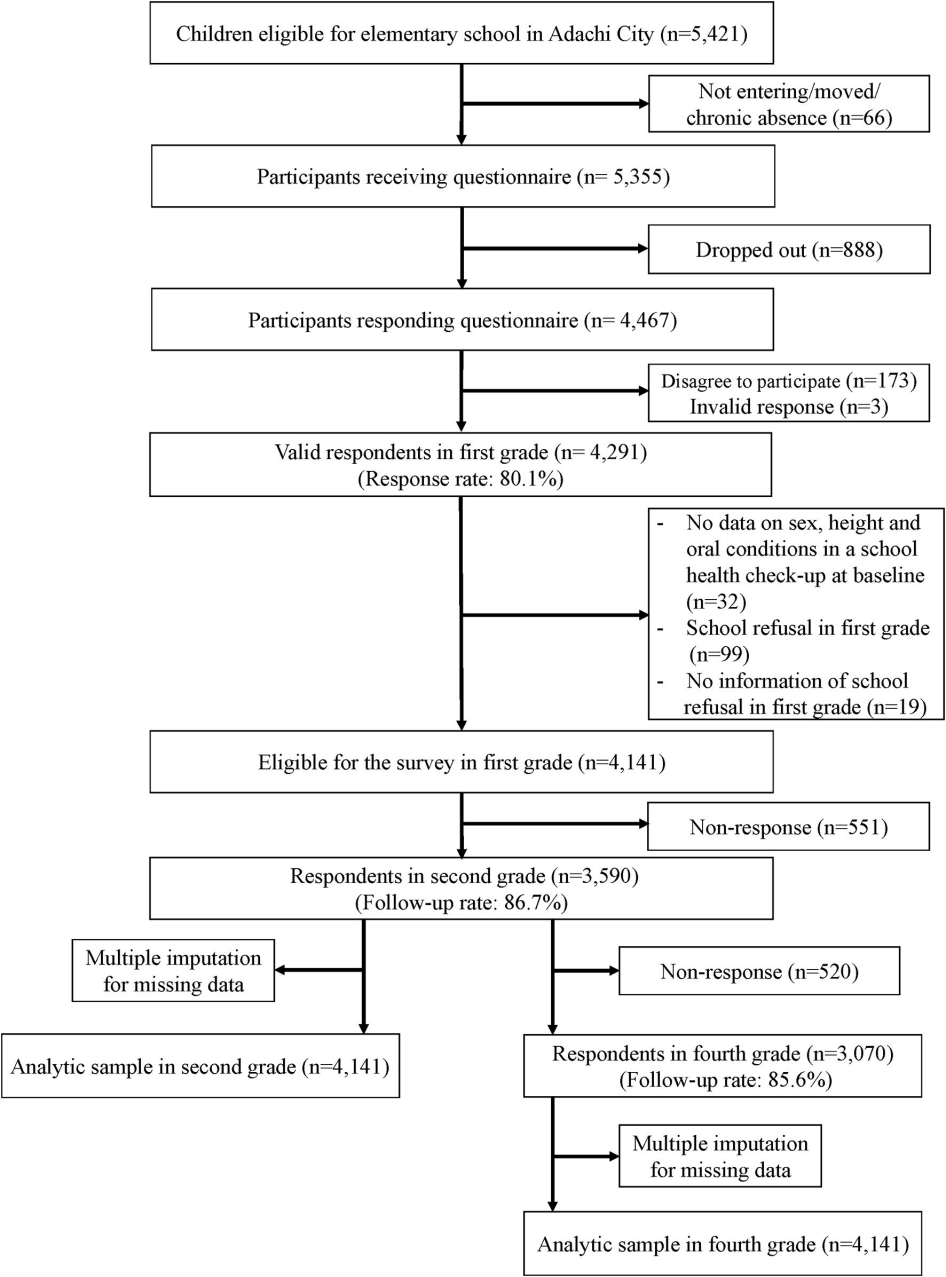
Flow chart of participants.

Data were from the Adachi Child Health Impact of Living Difficulty (A-CHILD) study conducted in 2015, 2016, and 2018 in Adachi City, Tokyo, Japan, which has 69 public elementary schools (28). In 2015, a self-reported questionnaire was distributed to all children in the first grade (6–7 years old) of all the elementary schools (*n* = 5,355, wave 1) in the city. Children took the questionnaire to home for their caregivers to fill out. The completed questionnaire was anonymously, but with the identification number, submitted in school (*n* = 4,467). A total of 4,291 caregivers gave informed consent (response rate: 80.1%). We excluded participants who had missing data at baseline (*n* = 32) on sex, height, and oral conditions in a school health check-up that all children in Adachi City are required to participate. Children who showed school refusal in the first grade and had no information about school refusal in the first grade were also excluded (*n* = 99 and 19, respectively). All the children (7–8 years old) were followed up in the second grade (*N* = 3,590, follow-up rate: 86.7%) in 2016 as wave 2. These children were followed up in the fourth grade (*n* = 3,070, follow-up: rate 85.6%) in 2018 as wave 3. A flow chart of the study participants is shown in Figure 1.

### Measurements

#### Parenting: Parent-Child Interaction

The parent–child interaction was assessed based on the frequency of nine types of parental interaction with their children in the first grade as follows: helping the child with schoolwork, playing with the child through physical exercise, playing video games with the child, playing card games or role-playing games with the child, talking with the child about school life, talking with the child about the news, talking about TV shows with child, cooking with the child, and going out with the child. Caregivers rated score to each question using a scale of 0 = “seldom,” 1 = “once or twice per month,” 2 = “once or twice per week,” 3 = “three or four times per week,” and 4 = “almost every day. We summed the parent–child interaction scores (the Cronbach's alpha = 0.72) and categorized them into tertile (1: low, 2: middle, and 3: high).

#### Child Maltreatment

Child maltreatment, including neglect, physical abuse, and psychological abuse, in the first grade was assessed by nine items adopted from the 17 items of Japanese child maltreatment scale (α = 0.77) (29, 30). Neglect was assessed by three items: “shut the child outside,” “do not feed the child,” and “leave the child alone in the house at night.” Physical abuse was measured by two items: “hit the body of the child (buttocks, hand, head, or face)” and “beat the child.” Psychological abuse was measured by three items: “yell at the child,” “insult the child repeatedly,” and “have a big fight in front of the child.” We did not use the item “ignore the child” because it can be a part of parental discipline and is difficult to classify clearly as a type of child maltreatment. A four-point Likert scale for each question was used (1 = “often,” 2 = “sometimes,” 3 = “rarely,” and 4 = “not at all”), and the caregivers scored. The responses were dichotomized, referring to expert review based on the severity and frequency of child maltreatment in Japan (31). As for the items of “hit the body of the child” and “yell at the child” are relatively prevalent, the response of “often” was classified as a “yes” response, and the responses “sometimes,” “rarely,” and “not at all” were classified as a “no” response. As for the items of “insult the child repeatedly” and “have a big fight in front of the child,” the responses “often” and “sometimes” were classified as a “yes” response, and the responses “rarely” and “not at all” as a “no” response. As for the items “beat the child,” “lock the child outside,” “do not feed the child,” and “leave the child alone in the house at night,” the responses “often,” “sometimes,” and “rarely” were classified as a “yes” response, whereas the response “not at all” as a “no” response. Then, when any items in each category of child maltreatment had a “yes” response once or more, we defined the category as “Yes” and dichotomized (1 = “Yes” and 0 = “No”).

#### School Refusal

Caregivers were asked whether their child was absent from school and, if so, the number of the days during the past 6 months since the beginning of the second and fourth grades, respectively. The caregivers also were asked the reason for school absence using the following categories: (1) illness or injury, (2) family reasons, (3) he/she did not want to go to school, and (4) other reasons. Then, we defined the response of three and the absence cases for more than 1 day as school refusal, and the response was dichotomized (1 = Yes, 0 = No).

#### Child Mental Health

The Emotional and behavior problems of children in the first grade, that is, emotional symptoms, conduct problems, hyperactivity/inattention, and peer problems, were assessed using the scales of total difficulties score from the Japanese version of the Strengths and Difficulties Questionnaire (SDQ) (32). The caregivers rated the score of the SDQ. The reliability and validity of the SDQ in Japanese children have been reported in previous research (33, 34).

In addition, the resilience of children in the first grade was assessed using the Children's Resilient Coping Scale (CRCS) (35). The scale consisted of eight items with high internal consistency (Cronbach's alpha = 0.80). The caregivers rated the resilience and coping behavior of their child from 0 (never) to 4 (very frequently). The score of the CRCS was calculated by summing up the score of the eight items and ranged from 0 to 32; a higher total score indicated a higher resilience.

#### Covariates

The sex of a child (boy or girl), birth order (no siblings, eldest, youngest, or middle), the marital status of the caregiver (married or single/others), and household income (<3.0, 3.0– <6.0, 6.0– <10.0, ≥10.0 million JPY; 110 JPY ≈ 1 USD) were used as covariates in the analysis. The mental health of the caregiver was assessed using the Japanese version of the Kessler 6 (K6) (36). It consists of six items about depression and anxiety rated on a five-point Likert scale. The total score of the six items was calculated (0–24); a higher score indicated a higher level of psychological distress. A score of 5–12 in the K6 was defined as the moderate psychological distress, and that of ≥13 was as the severe psychological distress (37).

### Statistical Analysis

We applied the multiple imputation approach under the assumption of the missing at random to minimize potential bias due to missing information. We generated 100 imputed datasets using the multiple imputation by chained equations procedure (MICE). The results were synthesized based on the Rubin's rule (38). Multivariate logistic regression models were fitted to calculate odds ratios (ORs) and 95% CIs for school refusal in the second and fourth grades, respectively. The following models were constructed: Model 1 was adjusted for the sex of a child, the marital status of the caregiver, birth order, household income, and K6 of the caregiver; in Model 2, all parenting variables (parent–child interaction with child and child maltreatment) were added to the Model 1; and Model 3 added child mental health (the total difficulties score of the SDQ and the score of the CRCS) to the Model 2; and Model 4 added school refusal in the second grade to the Model 3 (only in the analysis of school refusal in the fourth grade). For sensitivity analysis, we further investigated the associations of parenting with school refusal by the number of school refusal days. We used STATA version 15.0 (Stata Corp LLC, College Station, TX, USA) for all analyses.

### Ethics Statement

This study was approved by the Ethics Committee at the National Center for Child Health and Development (Study ID: 1147) and Tokyo Medical and Dental University (Study ID: M2016-284).

## Results

Table 1 shows the demographic characteristics of the participants in the first grade classified by school refusal status in the second grade. The prevalence of school refusal in the second grade was 1.8% (*n* = 64). As for the sex of a child, boys refused school more than girls in the second grade (56.3 and 43.8%, respectively). As for parenting, more children of school refusal showed the lower tertile of the parent–child interaction than those of non-school refusal (45.3 vs. 36.0%; *p* = 0.32). Similarly, regarding child maltreatment, more children of school refusal in the second grade experienced neglect (17.2 vs. 13.1%; *p* = 0.35), physical abuse (21.9 vs. 11.9%; *p* = 0.018), and psychological abuse (43.8 vs. 29.9%; *p* = 0.020) than children of non-school refusal.

**Table 1:** Demographic characteristics of the participants in the first grade by school refusal status in the second grade (*N* = 4,141).

	**Total**		**No school refusal** **(*****N*** **= 3,526)**		**School refusal** **(*****N*** **= 64)**		**Missing** **(*****N*** **= 551)**	
**Variables**	***n***	**%**	***n***	**%**	***n***	**%**	***n***	**%**
**Sex**								
Boys	2,121	51.2	1,804	51.2	36	56.3	281	51.0
Girls	2,020	48.8	1,722	48.8	28	43.8	270	49.0
Missing	0	0.0	0	0.0	0	0.0	0	0.0
**Marital status**								
Married	3,675	88.8	3,176	90.1	55	85.9	444	80.6
Single/others	359	8.7	268	7.6	9	14.1	82	14.9
Missing	107	2.6	82	2.3	0	0.0	25	4.5
**Birth order**								
No siblings	866	20.9	738	20.9	15	23.4	113	20.5
Eldest (having younger sibling)	1,315	31.8	1,130	32.1	23	35.9	162	29.4
Youngest (having elder sibling)	1,512	36.5	1,286	36.5	18	28.1	208	37.8
Middle (having both elder and younger sibling)	448	10.8	372	10.6	8	12.5	68	12.3
Missing	0	0.0	0	0.0	0	0.0	0	0.0
**Household income (million yen)**								
<3.0	460	11.1	365	10.4	10	15.6	85	15.4
3.0– <6.0	1,661	40.1	1,413	40.1	31	48.4	217	39.4
6.0– <10.0	1,256	30.3	1,103	31.3	15	23.4	138	25.1
≥10.0	354	8.6	310	8.8	4	6.3	40	7.3
Missing	410	9.9	335	9.5	4	6.3	71	12.9
**K6**								
<5	2,927	70.7	2,525	71.6	34	53.1	368	66.8
5– <13	977	23.6	829	23.5	21	32.8	127	23.1
≥13	182	4.4	136	3.9	9	14.1	37	6.7
Missing	55	1.3	36	1.0	0	0.0	19	3.5
**Parenting**								
**Parent–child interaction**								
Low	1,523	36.8	1,269	36.0	29	45.3	225	40.8
Middle	1,441	34.8	1,257	35.7	19	29.7	165	30.0
High	1,145	27.7	981	27.8	16	25.0	148	26.9
Missing	32	0.8	19	0.5	0	0.0	13	2.4
**Neglect**								
No	3,543	85.6	3,035	86.1	53	82.8	455	82.6
Yes	547	13.2	460	13.1	11	17.2	76	13.8
Missing	51	1.2	31	0.9	0	0.0	20	3.6
**Physical abuse**								
No	3,575	86.3	3,071	87.1	50	78.1	454	82.4
Yes	513	12.4	421	11.9	14	21.9	78	14.2
Missing	53	1.3	34	1.0	0	0.0	19	3.5
**Psychological abuse**								
No	2,818	68.1	2,436	69.1	36	56.3	346	62.8
Yes	1,264	30.5	1,054	29.9	28	43.8	182	33.0
Missing	59	1.4	36	1.0	0	0.0	23	4.2
**Child mental health**								
**SDQ**								
Total difficulties score (mean, SD)	9.8 (5.3)	–	9.7 (5.2)	–	12.1 (6.5)	–	–	–
**CRCS**								
Total score (mean, SD)	21.2 (4.9)	–	21.3 (4.8)	–	18.8 (5.7)	–	–	–

Table 2 shows the demographic characteristics of the participants in the first grade classified by school refusal status in the fourth grade. The prevalence of school refusal in the fourth grade was 2.0% (*n* = 60). As for the sex of a child, boys accounted for 61.7% of school refusal cases in the fourth grade. As for parenting, more children of school refusal showed the lower tertile of the parent–child interaction (40.0 vs. 35.5%; *p* = 0.49). Similarly, regarding child maltreatment, children of school refusal showed more neglect (16.7 vs. 13.1%; *p* = 0.44) and physical abuse (13.3 vs. 11.5%; *p* = 0.68), compared with children of non-school refusal. Psychological abuse was observed more in children of non-school refusal than in those of school refusal (30.1 vs. 26.7%; *p* = 0.57). The proportion of children refusing to go to school in the second grade was 15.0% among school refusal group in the fourth grade, which was higher than children of non-school refusal (15 vs. 1.3%; *p* < 0.001).

**Table 2:** Demographic characteristics of the participants in the first grade by school refusal status in the fourth grade (*N* = 4,141).

	**Total**		**No school refusal** **(*****N*** **= 3,010)**		**School refusal** **(*****N*** **= 60)**		**Missing** **(*****N*** **= 1,071)**	
**Variables**	***n***	**%**	***n***	**%**	***n***	**%**	***n***	**%**
**Sex**								
Boys	2,121	51.2	1,512	50.2	37	61.7	572	53.4
Girls	2,020	48.8	1,498	49.8	23	38.3	499	46.6
Missing	0	0.0	0	0.0	0	0.0	0	0.0
**Marital status**								
Married	3,675	88.8	2,750	91.4	44	73.3	881	82.3
Single/Others	359	8.7	195	6.5	13	21.7	151	14.1
Missing	107	2.6	65	2.2	3	5.0	39	3.6
**Birth order**								
No siblings	866	20.9	622	20.7	16	26.7	228	21.3
Eldest (having younger sibling)	1,315	31.8	980	32.6	17	28.3	318	29.7
Youngest (having elder sibling)	1,512	36.5	1,102	36.6	21	35.0	389	36.3
Middle (having both elder and younger sibling)	448	10.8	306	10.2	6	10.0	136	12.7
Missing	0	0.0	0	0.0	0	0.0	0	0.0
**Household income (million yen)**								
<3.0	290	9.6	8	13.3	162	15.1	460	11.1
3.0– <6.0	1,224	40.7	23	38.3	414	38.7	1,661	40.1
6.0– <10.0	950	31.6	16	26.7	290	27.1	1,256	30.3
≥10.0	274	9.1	4	6.7	76	7.1	354	8.6
Missing	272	9.0	9	15.0	129	12.0	410	9.9
**K6**								
<5	2,927	70.7	2,174	72.2	34	56.7	719	67.1
5–13	977	23.6	708	23.5	17	28.3	252	23.5
≥13	182	4.4	103	3.4	8	13.3	71	6.6
Missing	55	1.3	25	0.8	1	1.7	29	2.7
**Parenting**								
**Parent–child interaction**								
Low	1,523	36.8	1,069	35.5	24	40.0	430	40.2
Middle	1,441	34.8	1,069	35.5	23	38.3	349	32.6
High	1,145	27.7	857	28.5	13	21.7	275	25.7
Missing	32	0.8	15	0.5	0	0.0	17	1.6
**Neglect**								
No	3,543	85.6	2,590	86.1	50	83.3	903	84.3
Yes	547	13.2	395	13.1	10	16.7	142	13.3
Missing	51	1.2	25	0.8	0	0.0	26	2.4
**Physical abuse**								
No	3,575	86.3	2,639	87.7	52	86.7	884	82.5
Yes	513	12.4	347	11.5	8	13.3	158	14.8
Missing	53	1.3	24	0.8	0	0.0	29	2.7
**Psychological abuse**								
No	2,818	68.1	2,083	69.2	44	73.3	691	64.5
Yes	1,264	30.5	897	29.8	16	26.7	351	32.8
Missing	59	1.4	30	1.0	0	0.0	29	2.7
**Child mental health**								
**SDQ**								
Total difficulties score (mean, SD)	9.6 (5.2)	–	9.5 (5.1)	–	10.9 (5.6)	–	–	–
**CRCS**								
Total score (Mean, SD)	21.3 (4.8)	–	21.4 (4.8)	–	20.3 (5.3)	–	–	–
**School refusal in the second grade**								
No	3,526	85.2	2,971	98.7	51	85.0	504	47.1
Yes	64	1.6	39	1.3	9	15.0	16	1.5
Missing	551	13.3	0	0.0	0	0.0	551	51.5

Table 3 shows the results of multivariate logistic regression analysis for school refusal in the second grade after multiple imputation. In the crude model, the parent–child interaction in the first grade was not associated with school refusal in the second grade. Physical abuse and psychological abuse in the first grade showed association with school refusal in the second grade (OR: 2.10, 95% CI: 1.16–3.82 and OR 1.82, 95% CI: 1.11–2.98, respectively). No association between neglect in the first grade and school refusal in the second grade was observed. In Model 1, the parent–child interaction and each category of child maltreatment in the first grade showed no association with school refusal in the second grade, after adjusting for the sex of the child, the marital status of the caregiver, siblings, income, and K6 of the caregiver. As for child mental health, the total difficulties score of the SDQ in the first grade was associated with school refusal in the second grade (OR: 1.05, 95% CI: 1.01–1.10). The score of the CRCS in the first grade showed a significant inverse association with school refusal in the second grade (OR: 0.92, 95% CI: 0.88–0.97). In both Model 2, including all parenting variables, and Model 3, including all parenting and child mental health variables, the parent–child interaction and child maltreatment in the first grade were not associated with school refusal in the second grade. In Model 3, the total difficulties score of the SDQ in the first grade showed no association with school refusal in the second grade. As for the CRCS score in the first grade, the association with school refusal in the second grade remained significant (OR: 0.93, 95% CI: 0.87–0.99).

**Table 3:** Association between parenting in the first grade and school refusal in the second grade after multiple imputation.

	**Crude**		**Model 1**		**Model 2**		**Model 3**	
	**OR**	**95% CI**	**OR**	**95% CI**	**OR**	**95% CI**	**OR**	**95% CI**
**Parenting**								
**Parent–child interaction**								
Low	ref	–	ref	–	ref	–	ref	–
Middle	0.67	0.38–1.19	0.71	0.40–1.26	0.72	0.40–1.29	0.82	0.46–1.49
High	0.71	0.38–1.32	0.78	0.42–1.46	0.81	0.43–1.52	1.05	0.54–2.05
**Neglect**								
No	ref	–	ref	–	ref	–	ref	–
Yes	1.34	0.70–2.59	1.12	0.57–2.21	1.01	0.51–2.02	0.99	0.50–1.99
**Physical abuse**								
No	ref	–	ref	–	ref	–	ref	–
Yes	2.10^*^	1.16–3.82	1.55	0.83–2.90	1.38	0.70–2.71	1.29	0.65–2.55
**Psychological abuse**								
No	ref	–	ref	–	ref	–	ref	–
Yes	1.82^*^	1.11–2.98	1.41	0.84–2.37	1.25	0.71–2.19	1.14	0.64–2.00
**Child mental health**								
SDQ: Total difficulties score	1.08^***^	1.04–1.13	1.05^*^	1.01–1.10	–	–	1.01	0.95–1.06
CRCS: Total score	0.90^***^	0.86–0.94	0.92^**^	0.88–0.97	–	–	0.93^*^	0.87–0.99

*** *p < 0.001*,

** *p < 0.01*,

* *p < 0.05*.

*OR, odds ratio*.

*Model 1: adjusted for sex, birth order, the marital status of the caregiver, household income, and K6 of the caregiver*.

*Model 2: added all parenting variables to Model 1*.

*Model 3: added child mental health (the SDQ and the CRCS) to Model 2*.

Table 4 shows the results of multivariate logistic regression analysis for school refusal in the fourth grade after multiple imputation. In both crude and Model 1, there was no association between the parent–child interaction and each category of child maltreatment in the first grade and school refusal in the fourth grade. Similarly, Models 2 and 3 showed no association of the parent–child interaction and all categories of child maltreatment in the first grade with school refusal in the fourth grade. As for child mental health, in the crude model, the total difficulties score of the SDQ in the first grade was associated with school refusal in the fourth grade (OR: 1.05, 95% CI: 1.01–1.11). In Models 1 and 3, the association of total difficulties score of the SDQ and school refusal showed no significance. The score of the CRCS in the first grade was not associated with school refusal in the fourth grade in all models. Furthermore, in Model 4, children with school refusal at the second grade showed 11 times greater risk of school refusal at the fourth grade, independent of covariates.

**Table 4:** Association between parenting in the first grade and school refusal in the fourth grade after multiple imputation.

	**Crude**		**Model 1**		**Model 2**		**Model 3**		**Model 4**	
	**OR**	**95% CI**	**OR**	**95% CI**	**OR**	**95% CI**	**OR**	**95% CI**	**OR**	**95% CI**
**Parenting**										
**Parent–child interaction**										
Low	ref	–	ref	–	ref	–	ref	–	ref	–
Middle	0.95	0.54–1.69	1.07	0.59–1.92	1.02	0.57–1.86	1.07	0.59–1.96	1.07	0.57–2.01
High	0.71	0.35–1.44	0.83	0.40–1.72	0.80	0.38–1.66	0.85	0.40–1.82	0.83	0.41–1.68
**Neglect**										
No	ref	–	ref	–	ref	–	ref	–	ref	–
Yes	1.24	0.63–2.45	1.03	0.51–2.07	1.12	0.55–2.28	1.09	0.53–2.23	1.07	0.50–2.32
**Physical abuse**										
No	ref	–	ref	–	ref	–	ref	–	ref	–
Yes	1.15	0.53–2.48	0.78	0.34–1.79	0.98	0.41–2.37	0.94	0.39–2.27	0.84	0.35–2.02
**Psychological abuse**										
No	ref	–	ref	–	ref	–	ref	–	ref	–
Yes	0.85	0.47–1.54	0.60	0.32–1.15	0.59	0.30–1.17	0.56	0.28–1.11	0.56	0.27–1.14
**Child mental health**										
SDQ: total difficulties score	1.05^*^	1.01–1.11	1.02	0.96–1.07	–	–	1.02	0.96–1.09	1.03	0.94–1.08
CRCS: Total score	0.96	0.91–1.00	0.98	0.93–1.04	–	–	0.99	0.93–1.05	1.01	0.94–1.08
**School refusal in the second grade**										
No	ref	–	ref	–	–	–	–	–	ref	–
Yes	13.68^***^	6.39–29.27	10.99^***^	4.84–24.91	–	–	–	–	11.7^***^	5.02–27.4

*** *p < 0.001*,

* *p < 0.05*.

*OR, odds ratio*.

*Model 1: adjusted for sex, birth order, the marital status of the caregiver, household income, and K6 of the caregiver*.

*Model 2: added all parenting variables to Model 1*.

*Model 3: added child mental health (the SDQ and the CRCS) to Model 2*.

*Model 4: added school refusal in the second grade to Model 3*.

The sensitivity analysis investigating the associations of parenting with school refusal by the number of school refusal days showed that parenting was not associated with school refusal regardless of the number of the days (see Supplementary Tables 1–5).

## Discussion

In this study, we examined whether parenting was prospectively associated with school refusal among elementary school children. We found no associations of both parent–child interaction and child maltreatment in the first grade with school refusal in the second and fourth grades. Our results suggest that the parent–child interaction may not have a preventive effect on later school refusal among elementary school children, and child maltreatment may not be an independent risk factor for school refusal.

To our knowledge, no empirical studies have examined the association between parenting and school refusal using a longitudinal dataset. Previous research reported that school refusal was associated with the mental health of the caregiver (39) and socioeconomic status (11) as home environmental factors. Then, our models in this study assessed the effect of parenting on school refusal, adjusted for individual factors including the mental health of children and home environmental factors, together with the marital status of caregiver and siblings affecting the child development (40–42). Accordingly, our findings suggest that other factors, that is, school-related factors, may be more directly associated with school refusal among elementary school children rather than individual, parental, and home environmental factors. Indeed, prior studies have indicated that school-related problems, such as relationships with peers and teachers, school climate, and academic achievement, have an association with school refusal, which are known as risk factors (39, 43, 44). Thus, school-related problems may be more likely to be associated with school refusal for elementary school children. Further longitudinal research is needed to investigate the potential causal relationship between school-related problems and school refusal.

Moreover, we found no significant association between the parent–child interaction and school refusal, which renders this study the first to report the effect of the parent–child interaction on school refusal among elementary school children. We focused on the extent of the parent–child interaction, using the measurement variable of the interaction, which broadly incorporated various types of parental interaction at home. Accordingly, our finding suggests that the extent and types of the parent–child interaction may not contribute to the prevention of later school refusal. However, exactly how parents interact with their children in daily activities, that is, the quality of parent–child interaction or parental attitudes, was not assessed in this study. Prior research has indicated that improving the quality of parent–child interaction, which is applied in therapy, has preventive effects on behavior problems of children, such as aggressive behavior (45) and emotional problems, such as depression and anxiety (46, 47), which are the frequent comorbid conditions for school refusal. Further, as an example of the interaction, previous studies have reported that the positive parent–child communication is associated with fewer depressive symptoms (48), behavior problems (13), life satisfaction (49), and development of problem-solving strategies (14). Hence, given the beneficial effects, the quality of parent–child interaction may affect school refusal. However, whether there is an association between the quality of parental interaction with children and school refusal remains unclear. Future studies focusing on the effect of the quality of the interaction on school refusal are required. These may allow to fully understand the association between parent–child interaction and school refusal.

Our study revealed that child maltreatment, including neglect, physical abuse, and psychological abuse, had no association with later school refusal. A number of previous studies have shown that child maltreatment impairs the emotional and social development of children (50), and maltreated children have more relationship problems with their peers at school, such as peer rejection (51, 52). In addition, child maltreatment is negatively associated with academic performance (26, 53). These findings suggest that child maltreatment may indirectly increase the risk of school refusal, mediated by these peer-relationship problems, and lower academic performance. Moreover, maltreated children tend to have difficulties in school adjustment (54). However, in this study, we did not find an association between child maltreatment and school refusal. Although the exact reason remains unclear, one possible explanation is that school may function as a place for maltreated children to escape from home, similar to third places for maltreated children (55). Further research is needed to longitudinally evaluate the association between child maltreatment and school refusal with a qualitative design revealing the mechanisms.

There are several limitations in this study. First, school refusal, parental involvement, and child maltreatment were assessed by the caregiver, which may give rise to common method bias. Second, for the child maltreatment measurement, the scale was not validated, though it was based on self-report scales that have been widely used in Japan (56). And we did not assess traditional parenting styles, i.e., authoritative, authoritarian, permissive, and neglectful parenting (57, 58), due to limited space in the questionnaire. Moreover, we assessed only the quantity of parent–child interaction, but not for the quality of parent–child interaction, which is more important for the child development (59, 60). Third, although this longitudinal study showed a high response and followed-up rate, response bias should be considered. Given the characteristics of the outcome in this study, parents with children who refused to go to school may be unwilling to participate in the survey. To address the potential bias, we carried out using multiple imputation. Fourth, parental demographic factors might influence measurements of child mental health. Prior research reported that the total difficulties mean score of the SDQ for children of non-responding parents showed higher than that for children of responding parents (61). Thus, further, teacher-rating may help to assess child mental health more objectively. Fifth, we did not assess the physical problems and predisposition of children. Previous studies reported that oral health problems were associated with school absences and academic performance (62). Further, children with autism spectrum traits have an increased risk of school refusal (63). These factors may affect school refusal among children. Sixth, we lacked information on the reasons for school refusal, including the school-related problems in this study. Seventh, we did not use validated scales to assess for school refusal such as the School Refusal Assessment Scale (SRAS-R) (64, 65).

Despite these limitations, our current longitudinal study demonstrated no association between parenting and school refusal among elementary school refusal in population-based data. The empirical findings of this study provide a new understanding of the mechanisms of school refusal and the functions of individual, parental, and home environmental factors. Future research should explore the underlying causes of school refusal and the types of parenting that could play a preventive role in school refusal among children.

In conclusion, our study identified that the parent–child interaction and child maltreatment in the first grade were not associated with school refusal in the second and fourth grades among elementary school children in Japan. Further investigation is strongly recommended to better understand the complex association between the risk and protective factors and school refusal.

## Data Availability Statement

The original contributions presented in the study are included in the article/Supplementary Material, further inquiries can be directed to the corresponding author/s.

## Ethics Statement

The studies involving human participants were reviewed and approved by Tokyo Medical and Dental University Ethics Commitee. Written informed consent to participate in this study was provided by the participants' legal guardian/next of kin.

## Author Contributions

YF contributed to conception, design, analysis, and interpretation and drafted and critically revised the manuscript. SD and AI contributed to conception, design, data acquisition, analysis, and interpretation and critically revised the manuscript. MO contributed to conception, design, and data acquisition and critically revised the manuscript. TF contributed to conception, design, data acquisition, analysis, interpretation and drafted and critically revised the manuscript. All authors gave final approval and agreed to be accountable for all aspects of the work.

## Conflict of Interest

The authors declare that the research was conducted in the absence of any commercial or financial relationships that could be construed as a potential conflict of interest.
