# Supplementary material for: Association Between Parenting and School Refusal Among Elementary School Children in Japan: Results From A-CHILD Longitudinal Study
Source: Front Pediatr. 2021 Mar 26;9:640780. doi: 10.3389/fped.2021.640780 (PMC8032934; doi:10.3389/fped.2021.640780)
Supplement: Supplementary file 1 [file Table_1.docx]

**Association Between Parenting and School Refusal Among Elementary School Children in Japan: Results From A-CHILD Longitudinal Study**

**Authors:**

Yoshifumi Fukuya, Takeo Fujiwara, Aya Isumi, Satomi Doi and Manami Ochi

**Supplementary Tables**

| **Supplement Table 1. Number of school refusal days in second and fourth grade** | | |
| --- | --- | --- |
| Number of school refusal days | Frequency (%) | |
|  | second grade (n=64) | fourth grade (n=55) |
| 1 | 38 (59.4) | 20 (36.4) |
| 2 | 17 (26.6) | 17 (30.9) |
| 3 | 3 (4.7) | 5 (9.1) |
| ≥4 | 6 (9.4) | 13 (23.6) |

| **Supplement Table 2. Demographic characteristics of the participants in the first grade and school refusal in second grade (N=4141)** | | | | | | | | | |
| --- | --- | --- | --- | --- | --- | --- | --- | --- | --- |
| Variables | | No School Refusal  (N=3526) | | School Refusal (N=64) | | | | Missing  (N=551) | |
|  |  |  |  | One day (N=38) | | ≥ two days (N=26) | |  |  |
| **Sex** | | | | | | | | | |
|  | Boys | 1804 | 51.2 | 21 | 55.3 | 15 | 57.7 | 281 | 51.0 |
|  | Girls | 1722 | 48.8 | 17 | 44.7 | 11 | 42.3 | 270 | 49.0 |
|  | Missing | 0 | 0 | 0 | 0 | 0 | 0 | 0 | 0 |
| **Marital status** | | | | | | | | | |
|  | Married | 3176 | 90.1 | 30 | 79.0 | 25 | 96.2 | 444 | 80.6 |
|  | Single/Others | 268 | 7.6 | 8 | 21.1 | 1 | 3.8 | 82 | 14.9 |
|  | Missing | 82 | 2.3 | 0 | 0 |  | 0 | 25 | 4.5 |
| **Siblings** | | | | | | | | | |
|  | No siblings | 738 | 20.9 | 8 | 21.1 | 7 | 26.9 | 113 | 20.5 |
|  | Eldest (having younger sibling) | 1130 | 32.1 | 17 | 44.7 | 6 | 23.1 | 162 | 29.4 |
|  | Youngest (having elder sibling) | 1286 | 36.5 | 10 | 26.3 | 8 | 30.8 | 208 | 37.8 |
|  | Middle (having both elder and younger sibling) | 372 | 10.6 | 3 | 7.9 | 5 | 19.2 | 68 | 12.3 |
|  | Missing | 0 | 0 | 0 | 0.0 | 0 | 0 | 0 | 0.0 |
| **Household income (million yen)** | | | | | | | | | |
|  | < 3.0 | 365 | 10.4 | 5 | 13.2 | 5 | 19.2 | 85 | 15.4 |
|  | 3.0-<6.0 | 1413 | 40.1 | 17 | 44.7 | 14 | 53.8 | 217 | 39.4 |
|  | 6.0-<10.0 | 1103 | 31.3 | 10 | 26.3 | 5 | 19.2 | 138 | 25.1 |
|  | ≥10.0 | 310 | 8.8 | 3 | 7.9 | 1 | 3.8 | 40 | 7.3 |
|  | Missing | 335 | 9.5 | 3 | 7.9 | 1 | 3.8 | 71 | 12.9 |
| **K6** | | | | | | | | | |
|  | <5 | 2525 | 71.6 | 21 | 55.3 | 13 | 50.0 | 368 | 66.8 |
|  | 5-<13 | 829 | 23.5 | 12 | 31.6 | 9 | 34.6 | 127 | 23.1 |
|  | ≥13 | 136 | 3.9 | 5 | 13.2 | 4 | 15.4 | 37 | 6.7 |
|  | Missing | 36 | 1 | 0 | 0.0 | 0 | 0 | 19 | 3.5 |
| **Parenting** | | | | | | | | | |
| **Parent-child interaction** | | | | | | | | | |
|  | Low | 1269 | 36 | 15 | 39.5 | 14 | 53.8 | 225 | 40.8 |
|  | Middle | 1257 | 35.7 | 12 | 31.6 | 7 | 26.9 | 165 | 30 |
|  | High | 981 | 27.8 | 11 | 29 | 5 | 19.2 | 148 | 26.9 |
|  | Missing | 19 | 0.5 | 0 | 0.0 | 0 | 0 | 13 | 2.4 |
| **Neglect** | | | | | | | | | |
|  | No | 3035 | 86.1 | 35 | 92.1 | 18 | 69.2 | 455 | 82.6 |
|  | Yes | 460 | 13.1 | 3 | 7.9 | 8 | 30.8 | 76 | 13.8 |
|  | Missing | 31 | 0.9 | 0 | 0.0 | 0 | 0 | 20 | 3.6 |
| **Physical abuse** | | | | | | | | | |
|  | No | 3071 | 87.1 | 32 | 84.2 | 18 | 69.2 | 454 | 82.4 |
|  | Yes | 421 | 11.9 | 6 | 15.8 | 8 | 30.8 | 78 | 14.2 |
|  | Missing | 34 | 1 | 0 | 0.0 | 0 | 0 | 19 | 3.5 |
| **Psychological abuse** | | | | | | | | | |
|  | No | 2436 | 69.1 | 22 | 57.9 | 14 | 53.8 | 346 | 62.8 |
|  | Yes | 1054 | 29.9 | 16 | 42.1 | 12 | 46.2 | 182 | 33 |
|  | Missing | 36 | 1 | 0 | 0.0 | 0 | 0 | 23 | 4.2 |
| **Child mental health** | |  |  |  |  |  |  |  |  |
| **SDQ** | | | | | | | | | |
|  | Total Difficulties Score (Mean, SD) | 9.7 | 5.2 | 11.8 | 6.4 | 12.6 | 6.7 | - | - |
| **CRCS** | | | | | | | | | |
|  | Total Score (Mean, SD) | 21.3 | 4.8 | 18.5 | 5.8 | 19.1 | 5.6 | - | - |

| **Supplement Table 3. Demographic characteristics of the participants in the first grade and school refusal in fourth grade (N=4136)** | | | | | | | | | |
| --- | --- | --- | --- | --- | --- | --- | --- | --- | --- |
| Variables | | No School Refusal  (N=3010) | | School Refusal (N=55) | | | | Missing  (N=1071) | |
|  |  |  |  | One day (N=20) | | ≥two days (N=35) | |  |  |
| **Sex** | | | | | | | | | |
|  | Boys | 1512 | 50.2 | 13 | 65.0 | 20 | 57.1 | 572 | 53.4 |
|  | Girls | 1498 | 49.8 | 7 | 35.0 | 15 | 42.9 | 499 | 46.6 |
|  | Missing | 0 | 0 | 0 | 0 | 0 | 0 | 0 | 0 |
| **Marital status** | | | | | | | | | |
|  | Married | 2750 | 91.4 | 14 | 70.0 | 27 | 77.1 | 881 | 82.3 |
|  | Single/Others | 195 | 6.5 | 4 | 20.0 | 8 | 22.9 | 151 | 14.1 |
|  | Missing | 65 | 2.2 | 2 | 10.0 | 0 | 0 | 39 | 3.6 |
| **Siblings** | | | | | | | | | |
|  | No siblings | 622 | 20.7 | 5 | 25.0 | 10 | 28.6 | 228 | 21.3 |
|  | Eldest (having younger sibling) | 980 | 32.6 | 6 | 30.0 | 9 | 25.7 | 318 | 29.7 |
|  | Youngest (having elder sibling) | 1102 | 36.6 | 7 | 35.0 | 13 | 37.1 | 389 | 36.3 |
|  | Middle (having both elder and younger sibling) | 306 | 10.2 | 2 | 10.0 | 3 | 8.6 | 136 | 12.7 |
|  | Missing | 0 | 0 | 0 | 0 | 0 | 0 | 0 | 0 |
| **Household income (million yen)** | | | | | | | | | |
|  | < 3.0 | 8 | 13.3 | 2 | 10.0 | 6 | 17.1 | 460 | 11.1 |
|  | 3.0-<6.0 | 23 | 38.3 | 7 | 35.0 | 15 | 42.9 | 1661 | 40.1 |
|  | 6.0-<10.0 | 16 | 26.7 | 4 | 20.0 | 11 | 31.4 | 1256 | 30.3 |
|  | ≥10.0 | 4 | 6.7 | 2 | 10.0 | 2 | 5.7 | 354 | 8.6 |
|  | Missing | 9 | 15 | 5 | 25.0 | 1 | 2.9 | 410 | 9.9 |
| **K6** | | | | | | | | | |
|  | <5 | 2174 | 72.2 | 11 | 55.0 | 23 | 65.7 | 719 | 67.1 |
|  | 5-<13 | 708 | 23.5 | 7 | 35.0 | 6 | 17.1 | 252 | 23.5 |
|  | ≥13 | 103 | 3.4 | 1 | 5.0 | 6 | 17.1 | 71 | 6.6 |
|  | Missing | 25 | 0.8 | 1 | 5.0 | 0 | 0 | 29 | 2.7 |
| **Parenting** | | | | | | | | | |
| **Parent-child interaction** | | | | | | | | | |
|  | Low | 1069 | 35.5 | 6 | 30.0 | 17 | 48.6 | 430 | 40.2 |
|  | Middle | 1069 | 35.5 | 8 | 40.0 | 13 | 37.1 | 349 | 32.6 |
|  | High | 857 | 28.5 | 6 | 30.0 | 5 | 14.3 | 275 | 25.7 |
|  | Missing | 15 | 0.5 | 0 | 0 | 0 | 0 | 17 | 1.6 |
| **Neglect** | | | | | | | | | |
|  | No | 2590 | 86.1 | 18 | 90.0 | 29 | 82.9 | 903 | 84.3 |
|  | Yes | 395 | 13.1 | 2 | 10.0 | 6 | 17.1 | 142 | 13.3 |
|  | Missing | 25 | 0.8 | 0 | 0 | 0 | 0 | 26 | 2.4 |
| **Physical abuse** | | | | | | | | | |
|  | No | 2639 | 87.7 | 18 | 90.0 | 30 | 85.7 | 884 | 82.5 |
|  | Yes | 347 | 11.5 | 2 | 10.0 | 5 | 14.3 | 158 | 14.8 |
|  | Missing | 24 | 0.8 | 0 | 0 | 0 | 0 | 29 | 2.7 |
| **Psychological abuse** | | | | | | | | | |
|  | No | 2083 | 69.2 | 14 | 70.0 | 26 | 74.3 | 691 | 64.5 |
|  | Yes | 897 | 29.8 | 6 | 30.0 | 9 | 25.7 | 351 | 32.8 |
|  | Missing | 30 | 1 | 0 | 0 | 0 | 0 | 29 | 2.7 |
| **Child mental health** | | | | | | | | | |
| **SDQ** | | | | | | | | | |
|  | Total Difficulties Score (Mean, SD) | 9.5 | 5.1 | 10.1 | 6.1 | 10.2 | 6.1 | - | - |
| **CRCS** | | | | | | | | | |
|  | Total Score (Mean, SD) | 21.4 | 4.8 | 19.5 | 5.6 | 20.1 | 6.6 | - | - |

| **Supplement Table 4. Association between parenting in first grade and school refusal in second grade after multiple imputation** | | | | | | | | | | | | | |
| --- | --- | --- | --- | --- | --- | --- | --- | --- | --- | --- | --- | --- | --- |
|  | **Crude** | | | | | | **Adjusted model** | | | | | | |
|  | One day | | More than two days | | | | One day | | | | More than two days | | |
|  | RRR | 95%Cl | RRR | | 95%Cl | | RRR | | 95%Cl | | RRR | | 95%Cl |
| **Parenting** |  |  |  | |  | |  | |  | |  | |  |
| **Parent-child interaction** |  |  |  | |  | |  | |  | |  | |  |
| Low | ref | - | ref | | - | | ref | | - | | ref | | - |
| Middle | 0.80 | 0.37- 1.71 | 0.51 | | 0.20- 1.26 | | 1.05 | | 0.47- 2.33 | | 0.56 | | 0.22- 1.43 |
| High | 0.93 | 0.43- 2.03 | 0.47 | | 0.17- 1.29 | | 1.52 | | 0.64- 3.59 | | 0.59 | | 0.20- 1.72 |
|  |  |  |  | |  | |  | |  | |  | |  |
| **Neglect** |  |  |  | |  | |  | |  | |  | |  |
| No | ref | - | ref | | - | | ref | | - | | ref | | - |
| Yes | 0.58 | 0.18- 1.92 | 2.76* | | 1.19- 6.42 | | 0.48 | | 0.14- 1.64 | | 1.82 | | 0.71- 4.68 |
|  |  |  |  | |  | |  | |  | |  | |  |
| **Physical abuse** |  |  |  | |  | |  | |  | |  | |  |
| No | ref | - | ref | | - | | ref | | - | | ref | | - |
| Yes | 1.44 | 0.62- 3.33 | 3.02* | | 1.29- 7.06 | | 0.91 | | 0.35- 2.36 | | 1.90 | | 0.69- 5.24 |
|  |  |  |  | |  | |  | |  | |  | |  |
| **Psychological abuse** |  |  |  | |  | |  | |  | |  | |  |
| No | ref | - | ref | | - | | ref | | - | | ref | | - |
| Yes | 1.72 | 0.90- 3.30 | 1.84 | | 0.85- 3.98 | | 1.25 | | 0.59- 2.68 | | 0.92 | | 0.35- 2.37 |
|  |  |  |  | |  | |  | |  | |  | |  |
| **Child mental health** |  |  |  | |  | |  | |  | |  | |  |
| **SDQ: Total Difficulties Score** | 1.07* | 1.02- 1.13 | 1.09** | | 1.03- 1.16 | | 0.99 | | 0.92- 1.06 | | 1.03 | | 0.95- 1.13 |
|  |  |  |  | |  | |  | |  | |  | |  |
| **CRCS: Total Score** | 0.90*** | 0.85- 0.95 | 0.92* | | 0.85- 0.99 | | 0.90 | | 0.83- 0.97 | | 0.98 | | 0.90- 1.07 |
| ***p<0.001, **p<0.01,*p<0.05 ; RRR=Relative risk ratio; Cl=confidence interval. Reference: non-school refusal group | | | | | | | | | | | | | |
| Adjusted model: including all Parenting and child mental health variables, child's sex, parental marital status, siblings, household income and caregiver's K6 | | | | | | | | | | | | | |
| **Supplement Table 5 Association between parenting in first grade and school refusal in fourth grade after multiple imputation.** | | | | | | | | | | | | | |
|  | **Crude** | | | | | **Adjusted model** | | | | | | | |
|  | One day | | More than two days | | | One day | | | | More than two days | | | |
|  | RRR | 95%Cl | RRR | 95%Cl | | RRR | | 95%Cl | | RRR | | 95%Cl | |
| **Parenting** |  |  |  |  | |  | |  | |  | |  | |
| **Parent-child interaction** |  |  |  |  | |  | |  | |  | |  | |
| Low | ref | - | ref | - | | ref | | - | | ref | | - | |
| Middle | 1.29 | 0.45- 3.74 | 0.84 | 0.42- 1.66 | | 1.63 | | 0.54- 4.88 | | 0.88 | | 0.42- 1.81 | |
| High | 1.24 | 0.41- 3.7 | 0.43 | 0.17- 1.09 | | 1.75 | | 0.55- 5.58 | | 0.48 | | 0.18- 1.27 | |
|  |  |  |  |  | |  | |  | |  | |  | |
| **Neglect** |  |  |  |  | |  | |  | |  | |  | |
| No | ref | - | ref | - | | ref | | - | | ref | | - | |
| Yes | 0.68 | 0.17- 2.71 | 1.3 | 0.52- 3.23 | | 0.61 | | 0.14- 2.60 | | 1.15 | | 0.43- 3.07 | |
|  |  |  |  |  | |  | |  | |  | |  | |
| **Physical abuse** |  |  |  |  | |  | |  | |  | |  | |
| No | ref | - | ref | - | | ref | | - | | ref | | - | |
| Yes | 1.01 | 0.25- 4.01 | 1.18 | 0.46- 3.03 | | 0.85 | | 0.19- 3.87 | | 0.99 | | 0.32- 3.08 | |
|  |  |  |  |  | |  | |  | |  | |  | |
| **Psychological abuse** |  |  |  |  | |  | |  | |  | |  | |
| No | ref | - | ref | - | | ref | | - | | ref | | - | |
| Yes | 0.96 | 0.38- 2.44 | 0.85 | 0.39- 1.83 | | 0.82 | | 0.28- 2.38 | | 0.52 | | 0.2- 1.33 | |
|  |  |  |  |  | |  | |  | |  | |  | |
| **Child mental health** |  |  |  |  | |  | |  | |  | |  | |
| **SDQ: Total Difficulties Score** | 1.03 | 0.95- 1.12 | 1.06* | 1.01- 1.13 | | 0.97 | | 0.87- 1.09 | | 1.05 | | 0.97- 1.13 | |
|  |  |  |  |  | |  | |  | |  | |  | |
| **CRCS: Total Score** | 0.94 | 0.86- 1.02 | 0.96 | 0.90- 1.03 | | 0.92 | | 0.83- 1.04 | | 1.02 | | 0.94- 1.11 | |
| *p<0.05; RRR=Relative risk ratio; Cl=confidence interval. Reference group: non-school refusal group | | | | | | | | | | | | | |
| Adjusted model: including all Parenting and child mental health variables, child's sex, parental marital status, siblings, household income and caregiver's K6 | | | | | | | | | | | | | |
